# Addition of Copper Chloride and Zinc Chloride to Liquid-Stored Pig Semen Reduces Bacterial Growth Without Impairing Sperm Quality

**DOI:** 10.3390/ijms27020773

**Published:** 2026-01-13

**Authors:** Judit Drago, Elia Bosch-Rué, Nasira Akrim, Marc Yeste, Jordi Ribas-Maynou

**Affiliations:** 1 Biotechnology of Animal and Human Reproduction (TechnoSperm), Institute of Food and Agricultural Technology, University of Girona, 17003 Girona, Spain; 2 Unit of Cell Biology, Department of Biology, Faculty of Sciences, University of Girona, 17003 Girona, Spain; 3 Unit of Cell Biology and Medical Genetics, Department of Cell Biology, Physiology and Immunology, Autonomous University of Barcelona, 08193 Bellaterra, Spain

**Keywords:** sperm, boar, preservation, contamination, antimicrobial

## Abstract

Bacterial contamination remains a challenge for multiple facets of modern life. While antibiotics are a primary tool for bacterial control, their overuse has accelerated the appearance of multidrug-resistant bacteria and raises global health concerns. In swine, semen is stored at 17 °C in extenders that contain antibiotics to prevent bacterial growth. Apart from the potential consequences for the female, the proliferation of bacteria in liquid-stored semen is associated with a decline in sperm quality, ultimately reducing farrowing rates and litter sizes. With the aim of reducing the use of antibiotics while keeping bacterial growth under control, we herein investigated whether metal ions could exert an antimicrobial effect without impairing sperm quality. Separate metal ions (Ag, silver sulfadiazine; Al, aluminum chloride; Zn, zinc chloride; and Cu, and cooper chloride) were added at different concentrations (100 μM, 300 μM, 500 μM, 1 mM, and 10 mM) to seminal doses, which were stored at 17 °C for 48 h. Motility, viability, and the intracellular levels of reactive oxygen species (ROS) were tested to determine their effects on sperm quality maintenance. In addition, ions were added to bacterial strains and to extended seminal samples to assess the minimum inhibitory concentration (MIC) and minimum bactericidal concentration (MBC). Results showed that, although silver sulfadiazine exerted an antimicrobial effect at all the concentrations tested, it also affected sperm quality negatively (*p* < 0.05). In contrast, aluminum chloride did not impair sperm quality but failed to inhibit bacterial growth at any of the tested concentrations (*p* > 0.05). Finally, 1 mM concentrations of copper and zinc chloride reduced microbial growth (*p* < 0.05) without affecting sperm quality. In spite of this, the inhibition of bacterial growth was not complete, thus suggesting that these two ions could contribute to reducing bacterial growth but should be combined with other strategies, such as a lower storage temperature and a decreased concentration of antibiotics. Further research is warranted to address whether copper and zinc chloride could have a synergistic effect when added together.

## 1. Introduction

Optimizing farrowing rates and litter sizes is crucial for the swine industry, as it contributes to food access for the world’s population and maximizes farmers’ profitability. In pigs, maintaining semen quality in good shape is crucial to ensure the success of artificial insemination (AI), which is the standard breeding method in this species. Although the genetic selection of the best individuals has been crucial in recent decades for reaching a high efficiency in livestock [1,2], the separation of where semen is collected and doses are produced from the location where sows are inseminated poses the challenge of setting appropriate preservation methods for a certain period. Such strategies must ensure that the sperm’s fertilizing ability is not impaired while stored [3]. In swine, AI is mostly conducted with semen stored at 17 °C. This contrasts with other farm animals, where cryopreserved sperm or semen stored at 4–5 °C are used, which can be explained by the plasma membrane composition of pig sperm and their sensitivity to cold shock [4]. While the formulation of semen extenders has improved in such a way that it is currently possible to store sperm for some days [5], the potential microbial growth that is more likely to occur at 17 °C than at 4–5 °C, and this can only be mitigated with the addition of antibiotics to preservation media [6].

Experienced technicians collect porcine ejaculates in a draught-free environment. Yet, semen is—by definition—a non-sterile physiological fluid, which is highly exposed to diverse internal and external contamination sources [7]. Internal sources comprise urogenital infections, whereas external sources are usually associated with handling of boars to collect the ejaculate [8,9]. The presence of bacteria, particularly those of the Enterobacteriaceae family, which can grow at 17 °C, may end up in bacteriospermia, an important drawback of semen preservation at this temperature [10]. In effect, bacteriospermia induces sperm agglutination, reduces sperm motility and survival, and damages the DNA [11,12,13,14]. Antibiotics are the most used molecules to prevent bacterial growth, as they block or interfere with the bacterial cell cycle. Many commercial extenders for pig semen contain antibiotics, the most common are streptomycin, gentamycin, penicillin, lycomycin, amikacin, and chloramphenicol [15], which is in agreement with European Union regulations (Regulation (EU) 2016/429 of the European Parliament and of the Council of 9 March 2016). In spite of this, the continued presence of antibiotics in preservation media results in the appearance of resistant bacteria. This causes significant concern, as the appearance of multidrug-resistant bacteria may severely impact health [16]. For this reason, as in the case of human medicine, alternative approaches have been suggested to prevent bacterial growth without employing antibiotics; such substitutes include antimicrobial peptides (APs), phytoextracts, nanoparticles, and metal ions [4].

Antimicrobial peptides have been shown to inhibit bacterial growth and have been suggested as substitutes for antibiotics [17]. Shaoyang et al. [18] found that 0.16 g·L^−1^ ε-polylysine could decrease by a half the amount of gentamycin needed to keep bacterial growth under control (from 0.250 g/L to 0.125 g/L) without impairing sperm motility, mitochondrial membrane potential, acrosome and plasma membrane integrity, or in vitro fertilization outcomes [18]. Schulze et al. [19] observed that supplementing a short-term free-antibiotic extender (Beltsville Thawing Solution, BTS) with antimicrobial peptides such as WFW, c-WWW (both cyclic hexapeptides), and MK5E (helical magainin II amide derivative) prevented bacterial growth without negatively affecting sperm quality or AI outcomes. Finally, Bussalleu et al. [12] reported that, despite not being as effective as antibiotics, the PMAP-37 peptide could mitigate bacterial growth with no adverse effects on sperm quality at the optimal concentration; high concentrations could, however, be detrimental to sperm.

Phyto-extracts such as essential oils also exhibit antibacterial effects, although they may be cytotoxic at high concentrations. Elmi et al. suggested that 0.4 mg mL^−1^ of combined *Melaleuca alternifolia* and *Rosmarinus officinalis* extracts could replace antibiotics in semen, as this concentration did not affect sperm quality [20]. While other plant-derived compounds, such as flavonoids, quinones from roots of species like *Alkanna tinctoria* and *Arnebia euchroma*, and terpenoids, also exhibit antimicrobial and antioxidant activity, whether they could be utilized to substitute antibiotics in semen has not been interrogated [21]. Other substances that could be tested in semen for their antimicrobial and antioxidant effects include phenolic compounds, such as tannins, cinnamic acids, phenolic acids, and phenylpropanoids; water-soluble alkaloids like quinazolines, isoquinazolines; and indole derivatives, including betalains and eumelanins [21].

Metal ions have also been proposed to exert antimicrobial properties [22], despite being barely studied as antibiotic substitutes for sperm preservation. At appropriate concentrations, metal ions can reduce microbial growth through multiple complementary mechanisms. First, they can bind to and disrupt bacterial cell membranes, leading to loss of membrane integrity and subsequent cell death. In addition, certain metal ions promote the generation of reactive oxygen species (ROS), which are highly reactive toward biomolecules and can induce oxidative damage to lipids, proteins, and DNA, partly through Fenton-type reactions. Finally, some metal ions are able to interact with essential biomolecules, such as enzymes or nucleic acids, thereby interfering with protein function and DNA structure and ultimately inhibiting vital bacterial metabolic processes [23]. In effect, silver, zinc, and copper have been revealed to have antimicrobial properties against *Staphylococcus aureus* and *Escherichia coli* growth, without damaging fibroblasts [24]. When associated with nanoparticles, metal ions have been found to affect bacteria but not sperm [25,26]. For instance, silver nanoparticles at a concentration between 0.4 and 10 mM have been reported to inhibit *Staphylococcus aureus* growth without affecting sperm viability or the ability of sperm to elicit capacitation [27].

Against this background, the present work sought to test the antibacterial activity of different concentrations of metal ions (Al, aluminum chloride; Cu, copper chloride; Zn, zinc chloride; and Ag, silver sulfadiazine) during liquid-storage of pig semen at 17 °C, while also interrogating if they have any detrimental effect on sperm quality.

## 2. Results

### 2.1. Effects of Metal Ion Treatments on Sperm Quality

**Figure 1:**
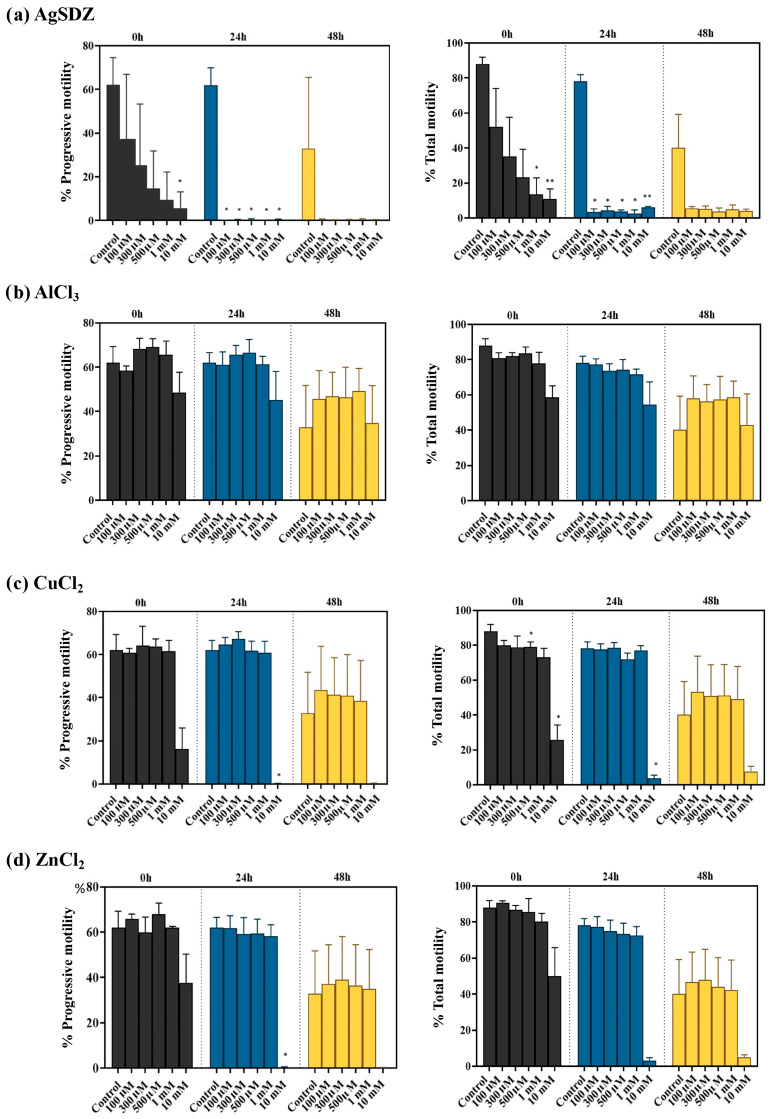
Percentages of progressive and total motility for silver sulfadiazine (**a**), aluminum chloride (**b**), copper chloride (**c**), and zinc chloride (**d**) at 100 µM, 300 µM, 500 µM, 1 mM, and 10 mM after 0, 24, and 48 h of storage at 17 °C. Bars indicate SEM. (*) Statistically significant differences compared to control samples (*p* ≤ 0.05). (**) Statistically significant differences compared to control samples (*p* ≤ 0.01).

Figure 1 shows the effect of metal ions on total and progressive sperm motility. Supplementary Tables S1 and S2 provide the specific values for sperm motility and kinematics, respectively. Immediately after addition (0 h), the treatment containing 10 mM AgSDZ reduced progressive and total motility (*p* < 0.05) compared to the control. Besides, at 0 h, a decreasing trend for total and progressive motility was observed for the highest concentrations (10 mM) of AlCl_3_, CuCl_2_, and ZnCl_2_, but this reduction was not statistically significant. No apparent effects were noticed for the other concentrations (100 μM–1 mM; Figure 1). After 24 h, all tested concentrations of AgSDZ (100 μM–10 mM) significantly (*p* < 0.01) reduced progressive and total motility. This contrasted with AlCl_3_ treatments, which were not different from the control (*p* > 0.05). In the case of CuCl_2_ and ZnCl_2_, the concentrations ranging between 100 μM and 1 mM had no effect, whereas that of 10 mM induced a severe reduction in progressive and total motility (*p* < 0.01). After 48 h of storage, samples treated with AgSDZ showed lower sperm motility (*p* < 0.01), those treated with AlCl_3_ exhibited no differences compared to the control (*p* > 0.05), and the ones treated with CuCl_2_ and ZnCl_2_ presented reduced motility only at the highest concentration tested (*p* < 0.01) (Figure 1).

**Figure 2:**
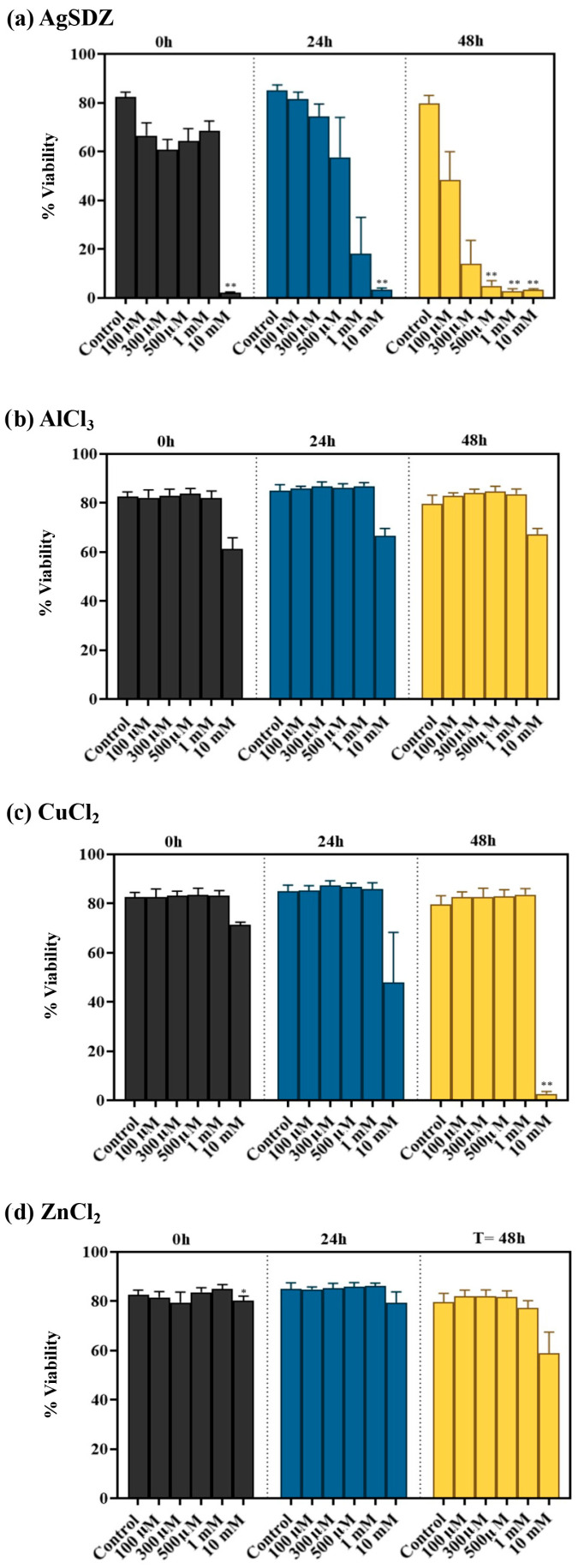
Percentage of viability for silver sulfadiazine (**a**), aluminum chloride (**b**), copper chloride (**c**), and zinc chloride (**d**) at 100 µM, 300 µM, 500 µM, 1 mM, and 10 mM after 0, 24, and 48 h of storage at 17 °C. Bars indicate SEM. (*) Statistically significant differences compared to control samples (*p* ≤ 0.05). (**) Statistically significant differences compared to control samples (*p* ≤ 0.01).

Figure 2 and Supplementary Table S3 depict how treating sperm with metal ions at 17 °C affected their viability. At the beginning of the experiment (0 h) and after 24 h of storage at 17 °C, the percentage of viable sperm in samples treated with 10 mM AgSDZ was significantly lower than in the control (*p* < 0.01), whereas the other ion treatments and concentrations had no impact (*p* > 0.05). After 48 h of storage, the percentage of viable sperm was significantly lower in samples treated with AgSDZ at a concentration equal to or higher than 300 μΜ. At 48 h, the sperm treated with 10 mM CuCl_2_ also exhibited lower viability (*p* < 0.01), whereas the other treatments and concentrations (i.e., AlCl_3_ and ZnCl_2_) did not significantly differ from the control.

**Figure 3:**
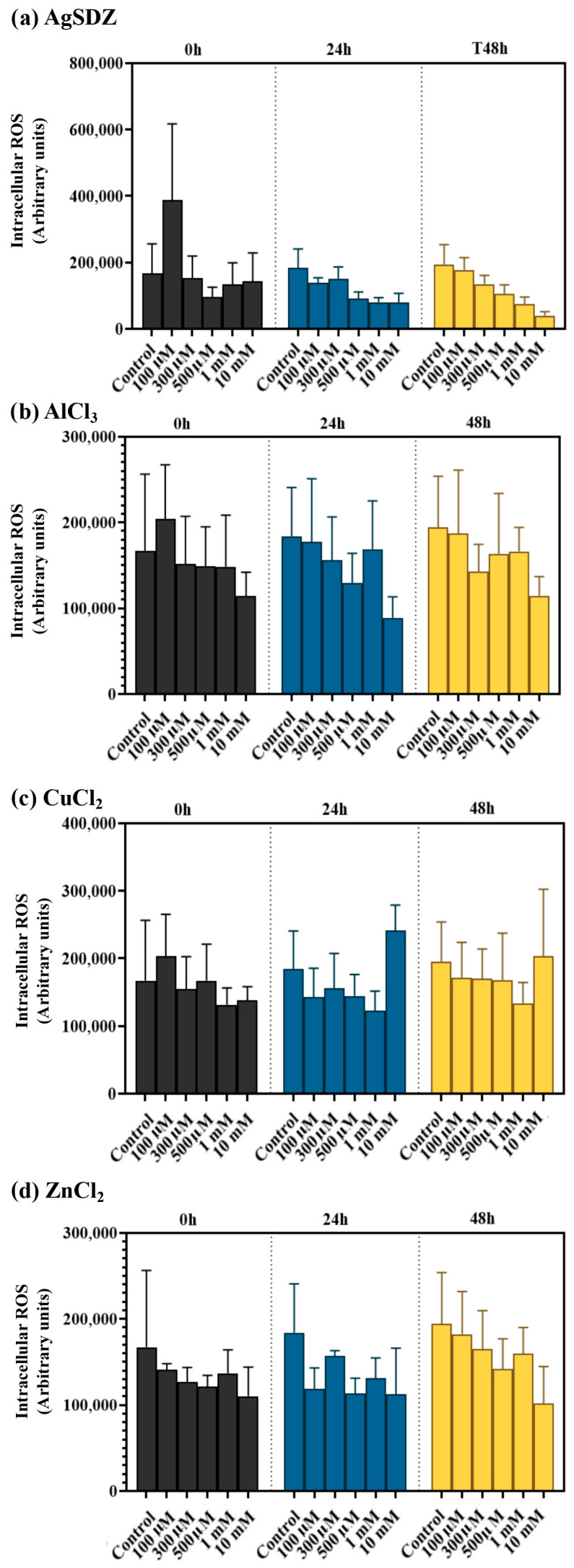
Intracellular reactive oxygen species (ROS; geometric mean of fluorescence intensity, arbitrary units) for silver sulfadiazine (**a**), aluminum chloride (**b**), copper chloride (**c**), and zinc chloride (**d**) at 100 µM, 300 µM, 500 µM, 1 mM, and 10 mM after 0, 24, and 48 h of storage at 17 °C. Bars indicate SEM. No statistically significant differences were found.

Finally, intracellular levels of ROS (Figure 3, Supplementary Table S4) were not altered by any ion treatment or concentration at 0 h, 24 h, or 48 h (*p* > 0.05).

### 2.2. Effect of Metal Ion Treatments on Bacterial Growth

**Figure 4:**
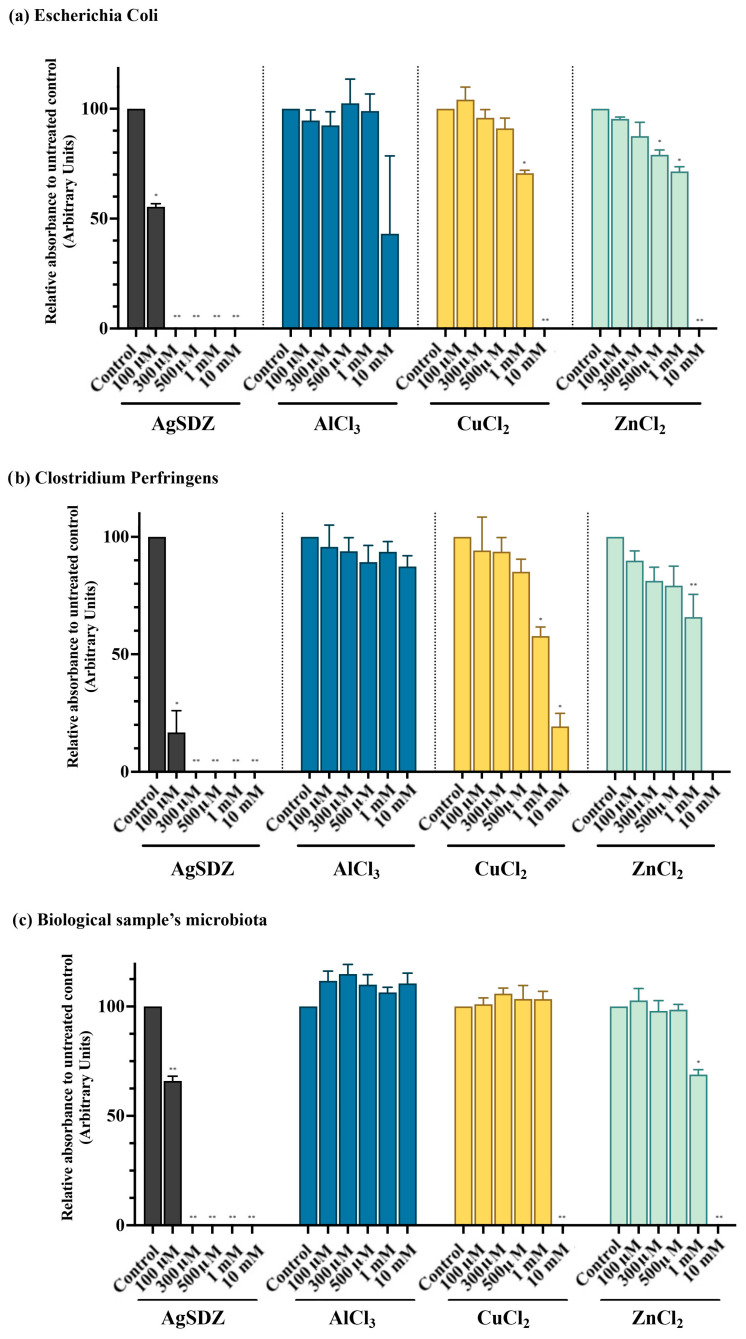
Bacterial growth measured at a wavelength of 600 nm (optical density, DO_600 nm_) for *Escherichia coli* (**a**), *Clostridium perfringens* (**b**), and the semen sample’s microbiota (**c**). For the two species, concentrations of 100 µM, 300 µM, 500 µM, 1 mM, and 10 mM of silver sulfadiazine (bars in grey), aluminum chloride (bars in electric blue), copper chloride (bars in yellow), and zinc chloride (bars in clear blue) are represented relative to the untreated control, which presented the maximum bacterial growth and was considered 100%. Bars indicate SEM. (*) Statistically significant differences compared to control samples (*p* ≤ 0.05). (**) Statistically significant differences compared to control samples (*p* ≤ 0.01).

Bacterial growth was tested in *Escherichia coli*, in *Clostridium perfringens*, and in the bacteria isolated from a seminal dose in order to calculate the minimum inhibitory concentration (MIC) for each ion. In addition, for those concentrations that inhibited the bacterial proliferation, the minimum bactericidal concentration (MBC) was calculated. All controls resulted in the lack of bacterial growth. Results of bacterial growth are shown in Figure 4. For those concentrations where bacterial growth was inhibited, the bactericidal effect is shown in Table 1.

As shown in Figure 4, treating bacteria with AgSDZ significantly (*p* < 0.01) inhibited bacterial growth at all the concentrations tested, such a reduction being greater than 50% at the minimum concentration evaluated (100 μM). The minimum inhibitory concentration was 250 μM for all species evaluated. Furthermore, whereas 300 μM AgSDZ was bactericidal for *Clostridium perfringens* (MBC), *Escherichia coli*, and the bacteria isolated from the semen sample needed a greater AgSDZ concentration (10 mM) to show the same effect.

As AlCl_3_ failed to inhibit bacterial growth at any of the concentrations tested (*p* > 0.05), its MIC and the MBC were suggested to be greater than 10 mM, which was the highest concentration tested in the present study (Table 1). CuCl_2_ inhibited the growth of *E. coli* and *Clostridium perfringens* at concentrations higher than 1 mM (*p* < 0.01), but needed a greater concentration (10 mM) to inhibit the proliferation of the bacteria isolated from the semen sample (*p* < 0.05). These results suggested that the MIC for CuCl_2_ was 10 mM, with the MBC higher than 10 mM.

Lastly, ZnCl_2_ showed inhibitory effects at 10 mM (*p* < 0.01) for the bacterial species tested, showing a dose-dependent reduction in bacterial growth from 250 mM to 10 mM. In addition, the ZnCl_2_ concentration required to decrease the growth of the bacteria isolated from the seminal dose was 1 mM. Based on these data, the MIC for ZnCl_2_ was 10 mM. Yet, as this concentration was not bactericidal for all the samples tested, the MBC was found to be greater than 10 mM.

### 2.3. Bacterial Growth on Extended Samples

**Figure 5:**
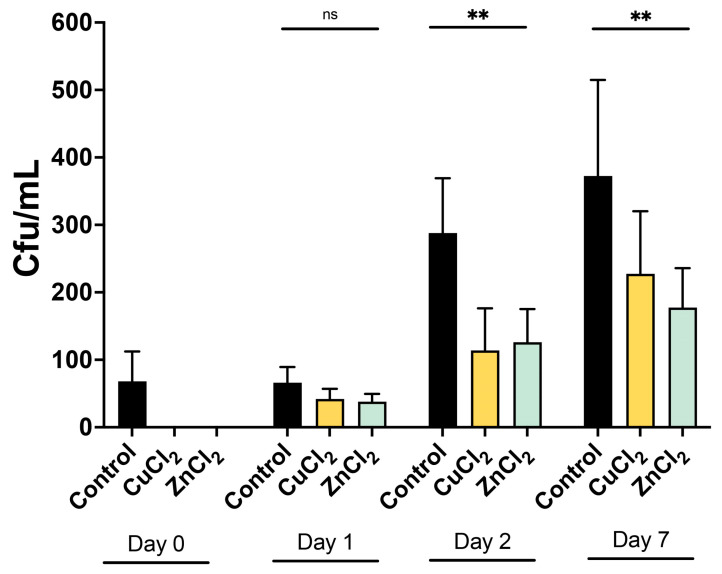
Colony-forming units per mL (CFU/mL) of the cultured semen after 24 h, 48 h, and 7 days of incubation with CuCl_2_ and ZnCl_2_, in comparison to the control extended without antibiotics. (ns) Values do statistically differ from control samples (*p* > 0.05). (**) Statistically significant differences compared to control samples (*p* ≤ 0.01).

Extended semen samples were evaluated for bacterial growth in the presence of 1 mM CuCl_2_ or ZnCl_2_ (Figure 5). A significant reduction in bacterial load, expressed as CFU/mL (*p* < 0.01), was observed after 48 h and 7 days of incubation with either ion compared to antibiotic-free controls. Specifically, CuCl_2_ reduced CFU/mL by 65 ± 24% after 48 h and by 63 ± 39% after 7 days of incubation. Similarly, ZnCl_2_ treatment resulted in reductions of 55 ± 23% and 64 ± 17% at 48 h and 7 days, respectively, relative to control samples extended without antibiotics.

## 3. Discussion

The presence of bacteria in seminal doses is a concern for artificial insemination in swine, as it hinders semen preservation, impairs sperm quality, and ultimately diminishes reproductive performance. For this reason, novel strategies for controlling bacteriospermia in pigs are needed [28,29]. In the present study, we interrogated whether treating seminal doses with metal ions could have a bacteriostatic or bactericidal effect without impairing sperm quality. Among the four compounds tested, we found that CuCl_2_ and ZnCl_2_ at 1 mM were able to reduce bacterial growth without impairing sperm motility, viability, or intracellular ROS. Although complete bacteriostatic and bactericidal activity was not observed at these concentrations, they could potentially allow for a reduction in the number and concentration of antibiotics in extended seminal doses.

Treating seminal doses with AgSDZ severely impaired sperm motility at various concentrations, but did not affect sperm viability or the levels of intracellular ROS. A fact worth noting is that this compound exerted a significant bacteriostatic and bactericidal effect, even at the lowest tested concentration. These results were not surprising, as the bactericidal properties of AgSDZ are well known [30] and, in fact, this compound is widely utilized as a topical antimicrobial agent, primarily employed in creams to control wound infections [31]. Indeed, silver ions are conjugated to nanoparticles or biomaterials like gelatin or thermoresponsive biogels, which are shown to be an effective treatment for resistant bacteria [24], to prevent infections in burn wounds [32], and to aid in the removal of biofilms [33]. Here, we found that the minimum inhibitory concentration for *E. coli* and *Clostridium perfringens* was 300 μM, and the minimum bacterial concentration was 10 mM. These findings are aligned with a previous study using silver nanoparticles, which reported a MIC and MBC of 579 μM against *Staphylococcus aureus* [34]. On the other hand, employing antimicrobial agents for semen preservation requires them not to be harmful to sperm quality. In somatic cells, silver compounds have been revealed as cytotoxic, as they induce apoptosis, increase intracellular ROS levels, and upregulate cytokine production in inflammatory cells [35]. Silver has also been flagged as a potential disruptor of spermatogenesis, affecting spermatogonia [36], and has been reported to reduce the motility of sheep sperm [37]. To the best of our knowledge, this is the first study demonstrating that treating pig sperm with a silver compound (AgSDZ) negatively affects sperm motility and viability, even at a concentration as low as 100 μM. Thus, despite its apparent antibacteriostatic and antibactericidal activity, the spermicidal side effects of AgSDZ limit its potential application for the control of bacteriospermia in pig semen, and should not be used for this purpose.

At the concentrations tested, AlCl_3_ did not negatively affect the motility, viability, or intracellular ROS levels of pig sperm, with all parameters remaining similar to the control. As no previous investigations have examined the toxicity of AlCl_3_ in mammalian sperm, it was unknown whether this compound could be toxic when added to semen. Other investigations found that individuals exposed to high aluminum concentrations experience testicular damage, including increased free radical production and apoptosis, leading to severe alterations during spermatogenesis and ultimately causing fertility impairment [38,39]. Aluminum has been proposed as an antibacterial agent [40] and has been described to attack bacterial ferredoxins containing [4Fe-4S] clusters, disrupting electron transfer and inducing ROS production in bacteria [23]. Yagaza et al. [41] showed that treating bacteria with 0.2 M AlCl_3_ caused bacterial cell wall degradation and cytoplasmic aggregation, whereas bacteria exposed to lower concentrations (up to 0.1 M) showed no changes [41]. In our study, we did not observe any bacteriostatic or bactericidal effect of AlCl_3_; however, based on the report mentioned above [41], it could be that the concentrations tested here were below the bactericidal range. Further research should thus address whether higher concentrations could be harmful to sperm.

Treating sperm with CuCl_2_ at concentrations up to 1 mM did not impair sperm quality, nor did it cause an increase in reactive oxygen species, suggesting that this compound is not cytotoxic for pig sperm at these levels. While no previous study evaluated the toxicity of CuCl_2_ in porcine sperm, treating their bovine counterpart at concentrations equal to or lower than 7.80 μM was not observed to negatively impact sperm quality, and it facilitates the activation of the cytochrome oxidase system [42]. Yet, the same study found that concentrations higher than 300 μM reduced sperm motility [42]. Similarly, treating sperm from *Colossoma macropomum*, a fish species, with concentrations of CuCl_2_ higher than 8 mg/L (~6 mM) was reported to impair sperm motility and fertilizing ability [43]. The differences between species and preservation media could explain these disparate results. In effect, porcine and bovine sperm differ in their antioxidant content and the composition of preservation media [44]. Furthermore, greater physiological differences exist between mammalian and fish sperm. Our results suggest that extended pig sperm could be more resilient to copper damage, probably due to the presence of antioxidants in sperm cells, seminal plasma, and even the extender [45]. Copper is an antimicrobial agent whose effectiveness against a number of microorganisms has been proven [46]. The antimicrobial mechanism of copper and other heavy metal ions is attributed to their toxic effects on bacterial membranes, which are disrupted when an interaction with the ion occurs. In addition, cooper induces oxidative stress in bacteria, which causes DNA base modifications, protein oxidations, and lipid oxidations, ultimately leading to bacterial death [47]. In our study, 1 mM CuCl_2_ impaired the growth of *E.coli* and *Clostridium perfringens*. The MIC was found to be 10 mM, and the MBC was greater than 10 mM, values that were below those observed in other experiments, where the MBC was reported to be 1.42 mM [48]. Yet, the bacterial species used in that study, *Staphylococcus epidermidis*, was different from the one used in the current work. Thanks to our experiments in extended semen, we found that microbial contamination is reduced after 48 h and after 7 days of incubation in 1 mM CuCl_2_ in comparison to semen extenders without antibiotics. Therefore, the addition of this compound, combined with a reduced amount of antibiotics, could be useful to prevent bacterial growth.

The results of ZnCl_2_ treatments closely matched those observed with CuCl_2_. Regarding sperm quality, only concentrations greater than 10 mM reduced motility and viability, which indicates that lower ZnCl_2_ concentrations could be compatible with sperm function. Previous research showed that the supplementation of pig sperm with ZnCl_2_ at 20 μg/mL and 50 μg/mL—equivalent to 147.7 μM and 366.7 μM, respectively—conveyed a protective effect to boar sperm compared to non-supplemented [49]. Also, zinc ions are known to be important for sperm chromatin integrity, flagellar function, motility, acrosomal exocytosis, and capacitation [50,51]. While zinc is also involved in bacterial cell proliferation, as part of metabolic pathways [52,53], it has a detrimental effect at high concentrations, which could be driven by oxidative mechanisms or direct interaction with bacterial walls [54,55,56]. In our study, ROS levels did not increase even after 48 h of incubation, which would suggest that liquid-stored pig sperm could withstand the oxidative-related damaging effects of zinc. Our data showed that, at 1 mM, ZnCl_2_ is able to reduce bacterial growth, both in selected strains and in extended semen incubated at 17 °C. Interestingly, Hernández-Sierra et al. [57] reported that zinc nanoparticles have a MIC of 500 μg/mL (7.65 mM) against *Streptococcus mutans*, which would be consistent with the MIC observed in this study. Hence, supplementing semen extenders with 1 mM ZnCl_2_ could contribute to preventing bacterial growth without altering sperm quality and could thus be used as a strategy to reduce the amount of antibiotics included in preservation media.

Finally, our strategy using 1 mM CuCl_2_ or 1 mM ZnCl_2_ was effective at reducing bacterial load in extended samples and in specific strains such as Escherichia coli and Clostridium perfringens. Nevertheless, we acknowledge that this study has limitations. Firstly, the samples used were handled following standard routines used in the AI industry, and although they exhibited measurable bacterial loads, these levels were relatively low. Our results are based on these observed levels, and it remains uncertain whether similar reductions would be observed in samples with higher bacterial loads. Second, a comprehensive evaluation of changes in the bacterial microbiota following incubation with these ions might allow us to determine whether the relative abundance of each bacterial family or genus is maintained or altered, and could identify specific genera exhibiting increased resistance to antimicrobial ions. In this regard, the evaluation of the effects of copper and zinc on Serratia and Klebsiella genera is of particular interest due to their well-documented detrimental effects on sperm, as ongoing efforts aimed at mitigating their impact [58].

## 4. Materials and Methods

Unless otherwise stated, all reagents were purchased from Sigma-Aldrich (Saint-Louis, MO, USA).

### 4.1. Experimental Design and Semen Samples

The present study was designed in three stages to address whether different concentrations of aluminum chloride (AlCl_3_), copper chloride (CuCl_2_), zinc chloride (ZnCl_2_), and silver sulfadiazine (AgSDZ) possess antimicrobial properties when semen is stored at 17 °C, and if such concentrations have an adverse effect on sperm quality. In the first subset of experiments, we tested the effects of the presence or absence of each compound at different concentrations on sperm motility, viability, and intracellular levels of reactive oxygen species (ROS) for a 48 h period at 17 °C. In the second subset of experiments, the same molecules were tested against representative bacterial species and the bacteria typically present in pig semen to determine the minimum inhibitory concentration (MIC). For those concentrations that were inhibitory for bacterial growth, we further evaluated the minimum bactericidal concentration (MBC). In the third subset of experiments, the antibacterial efficacy of the selected ions (Cu and Zn) was evaluated directly in extended semen samples stored for 24 h, 48 h, and 7 days at the MIC and MBC concentrations (1 mM). Bacterial growth was assessed by culturing aliquots in rich medium.

For the first and second subsets of experiments, we used three seminal doses from three sexually mature Pietrain boars, and for the third subset of experiments, we used five different seminal doses from sexually mature Pietrain boars. All samples were provided by a local farm operating under standard commercial conditions (Servicios Genéticos Porcinos, S.L.; Roda de Ter, Spain) and were obtained using the gloved-hand method. In all experimental subsets, semen samples were diluted to a final concentration of 33 × 10^6^ sperm/mL in a commercial extender that did not contain antibiotics (Androstar^®^ Plus without antibiotics, Minitübe, Tiefenbach, Germany) and subsequently handled following the same procedures routinely applied in the industry, in order to mimic the bacterial loads naturally present in these samples.

The AI center that provided the samples performed all the procedures that involved animals following the Directive 2010/63/EU of the European Parliament and of the Council of 22 September 2010, the Animal Welfare Law issued by the Regional Government of Catalonia, and the current regulation on Health and Biosafety issued by the Department of Agriculture, Livestock, Food and Fisheries, Generalitat de Catalunya, Spain. As animals were not directly manipulated and semen was obtained for commercial purposes, the study did not require the approval of any specific ethics committee.

### 4.2. Evaluation of the Effect of Ions on Sperm Quality

Aluminum chloride, copper chloride, zinc chloride, or silver sulfadiazine were added at different concentrations (100 μmol/L, 300 μmol/L, 500 μmol/L, 1 mmol/L, and 10 mmol/L) to semen samples, which were then stored at 17 °C for 48 h. A negative control without the addition of these compounds was also included. Sperm motility and viability, and intracellular levels of reactive oxygen species were evaluated at 0 h, 24 h, and 48 h.

#### 4.2.1. Motility Evaluation

Sperm motility was assessed using a Computer-Assisted Sperm Analysis (CASA) system (ISAS v1, Proiser, S.L.; València, Spain), coupled to an Olympus BX41 microscope (Olympus; Tokyo, Japan), and under a negative phase-contrast objective. Before evaluation, samples were incubated at 38 °C for 15 min. Briefly, 3 μL of each sample was subsequently loaded into a pre-warmed 20 μm Leja chamber slide (Leja Products BV; Nieuw Vennep, The Netherlands) and observed under the microscope (100× magnification). Different fields were recorded at a frame rate of 25 images per second, evaluating at least 1000 sperm. Two technical replicates were examined for each treatment. The software provided information regarding total motile sperm (%), sperm with progressive motility (%); sperm with rapid (>30 μm/s), medium (15–30 μm/s) and slow (0–15 μm/s) motility (%); curvilinear velocity (VCL; sequential sperm progression along the whole trajectory; μm/s); straight-line velocity (VSL; straight sperm trajectory per unit of time; μm/s); average path velocity (VAP; sperm trajectory per unit of time; μm/s); linearity coefficient (LIN = VSL/VCL × 100; %); straightness coefficient (STR = VSL/VAP × 100; %); wobble coefficient (WOB = VAP/VCL × 100; %); mean amplitude of lateral head displacement (ALH; amplitude of the lateral oscillatory movement of the sperm head around the mean trajectory; μm); and frequency of head displacement (BCF; the number of sperm head lateral oscillatory movements around the mean trajectory per unit of time; Hz).

#### 4.2.2. Flow Cytometry

Sperm viability and intracellular levels of reactive oxygen species were evaluated using a CytoFLEX flow cytometer (Beckman Coulter, Fullerton, CA, EUA) equipped with violet, blue, and red lasers (405 nm, 488 nm, and 637 nm). The flow cytometer fluorescence intensity was calibrated daily using Cytoflex Daily QC Fluorospheres (Beckman Coulter, Fullerton, CA, USA), according to the manufacturer’s instructions. Staining with the corresponding concentrations of each fluorochrome was conducted in 200 μL of PBS containing sperm at a concentration of 1 × 10^6^ sperm/mL, and acquisition was set at a constant flow-rate between 10 and 60 μL/s. Results were analyzed using the CytExpert software v2.2 (Beckman Coulter, Fullerton, CA, EUA). Only particles with similar FSC-A and FSC-H values were gated in, thus excluding doublets from the analysis. The flame-shaped population corresponding to sperm cells was gated in the FSC-A/SSC-A dot plot.

For the assessment of sperm viability, samples were incubated with 31.5 nmol/L of SYBR-14 and 7.6 μmol/L of propidium iodide (PI) at 38 °C for 10 min. Both fluorochromes were excited at 488 nm, and the fluorescence was collected through the FITC channel (525/40) for SYBR-14, and the PC5.5 channel (690/50) for PI. Four populations were detected, corresponding to viable sperm (SYBR-14^+^/PI^−^), non-viable sperm (SYBR-14^−^/PI^+^ and SYBR-14^+^/PI^+^), and debris particles (SYBR-14^−^/PI^−^). Percentages of viable sperm were recalculated after excluding debris particles.

Evaluation of intracellular reactive oxygen species was conducted following co-staining with 2′,7′-dichlorodihydrofluorescein diacetate (H_2_DCFDA) and PI. H_2_DCFDA reacts with ROS, generating dichlorofluorescein (DCF), which emits green fluorescence when excited at 488 nm. Sperm were incubated in 100 μmol/L H2DCFDA and 5.6 μmol/L PI at 38 °C in the dark for 20 min. Four populations were distinguished in dot-plots: (i) non-viable sperm with a high content of reactive oxygen species (ROS) (DCF^+^/PI^+^), (ii) non-viable sperm with a low content of ROS (DCF^−^/PI^+^), (iii) viable sperm with a low content of ROS (DCF^−^/PI^−^), and (iv) viable sperm with a high content of ROS (DCF^+^/PI^−^). The fluorescence of DCF was collected through the FITC channel (525/40), whereas that of PI was collected by the PC5.5 channel (690/50). The percentages of the four populations were recalculated after excluding the debris particles as determined by the SYBR14^+^/PI^−^ staining.

### 4.3. Evaluation of Antimicrobial Properties

In order to assess whether ions had antimicrobial properties, the minimum inhibitory concentration (MIC) and the minimum bactericidal concentration (MBC) for each treatment were determined. Whereas the former was used to evaluate the concentrations inhibiting bacterial growth, the latter allowed determining which concentrations that prevented growth also caused bacterial death.

Both MIC and the MBC were examined in two commercial pure bacterial species that are usually found in pig semen (*Escherichia coli* and *Clostridium perfringens*), and in bacteria isolated from a semen sample that could be reflective of the fluid’s microbiota. In the third case, to isolate bacteria from a semen sample, seminal doses obtained without antibiotics were incubated at 37 °C for 12 h. The sample was subsequently centrifuged at 600× *g* for 5 min to separate sperm from bacteria, which remained in the supernatant. The supernatant was mixed 1:1 (*v*:*v*) with glycerol, homogenized, and stored in a Nalgene freezing container (Nalge Nunc Int. Corp., New York, NY, USA) at −80 °C. The day after, cryovials were transferred to a −80 °C freezer.

#### 4.3.1. Determination of the Minimum Inhibitory Concentration (MIC)

The MIC was determined as described in Balouiri et al. [16]. Frozen bacterial stocks were scraped from cryovials, resuspended in 5 mL of Mueller–Hinton Broth, and incubated overnight at 37 °C in agitation. After incubation, bacterial suspensions were adjusted to a 0.5 McFarland Standard (1–2 × 10^8^ CFU/mL), using a spectrophotometer (Epoch Spectrophotometer, Beckman Coulter, Fullerton, CA, USA).

To ensure uniform bacterial density, serial dilutions were prepared until an optical density at 600 nm (DO_600 nm_) of 0.08 was achieved.

One mL of the adjusted bacteria suspensions (1–2 × 10^8^ CFU/mL) was incubated with aluminum chloride, copper chloride, zinc chloride, and silver sulfadiazine at the following concentrations: 100 μM, 300 μM, 500 μM, 1 mM, and 10 mM. A sterility control in which the different ion treatments were conducted in the absence of bacteria. All samples were incubated at 37 °C for 24 h in constant agitation. Negative controls were streaked in Mueller–Hinton agar and incubated at 37 °C overnight to check bacterial growth [17]. To assess bacterial growth, OD_600_ was measured post-incubation using spectrophotometry (Epoch Spectrophotometer, Beckman Coulter, Fullerton, CA, USA). Data were normalized against blank solutions (ion treatments without bacteria). The bacterial load in the negative control was considered 100%. Ion concentrations that showed complete inhibition or significant reduction in bacterial growth were identified. The lowest concentration with no detectable bacterial growth was recorded as the MIC.

#### 4.3.2. Determination of the Minimum Bactericidal Concentration (MBC)

Among the ion treatments that inhibited bacterial growth, the minimum bactericidal ion concentration was determined. This variable corresponded to the minimum concentration that caused the absence of colonies in Petri dishes. Briefly, 100 μL of each bacterial-ion mixture—at the concentrations where there was no bacterial growth—was seeded onto Mueller–Hinton agar plates and incubated at 37 °C for 24 h. Colony formation was assessed the next day. The lowest concentration that resulted in no colony growth was considered the MBC.

#### 4.3.3. Determination of Bacterial Growth in Rich Medium

To evaluate whether microbial growth was prevented in extended semen samples, five boar semen samples, extended without antibiotics, were incubated with 1 mM CuCl_2_ or 1 mM ZnCl_2_ for 24 h, 48 h, and 7 days. At each time point and for each treatment, 100 μL of each sample was plated onto Luria–Bertani agar plates and incubated at 37 °C for 24 h. Colony formation was assessed the following day, and bacterial counts were expressed as colony-forming units per milliliter (CFU/mL).

### 4.4. Statistical Analysis

Statistical analyses were conducted using the Statistics Package for Social Sciences (SPSS) ver. 25.0 (IBM Corp.; Armonk, NY, USA), and graphs were elaborated with the GraphPad Prism ver. 8 (GraphPad Software, La Jolla, CA, USA). Normal distribution and homogeneity of variables were checked with the Shapiro–Wilk and Levene tests. As even after linear transformation, the data did not meet the parametric assumptions, non-parametric tests were run. To evaluate the effects of metal ions on sperm quality, a non-parametric two-way ANOVA (Scheirer-Ray-Hare test) was conducted, with the time (0 h, 24 h, and 48 h) and ion treatment/concentration as factors. To assess the effects of treating bacteria for 48 h with ion treatments, the Kruskal–Wallis test was used. Pair-wise comparisons were made with the Mann–Whitney U test. For all tests, the level of statistical significance was set at *p* < 0.05.

## 5. Conclusions

In summary, our study evaluated the antimicrobial properties of four different metal ion compounds, assessing their effects on both sperm quality and bacterial growth. Data presented here suggest that supplementing extended pig semen with 1 mM CuCl_2_ or 1 mM ZnCl_2_ could help mitigate bacterial growth, potentially allowing a reduction in the concentration of antibiotics used. Further studies should evaluate whether combining Cu and Zn could have a synergistic effect while decreasing bacterial growth, as well as assessing the specific effects on other highly resistant bacterial strains such as *Serratia* spp. and *Klebsiella* spp.

## Figures and Tables

**Table 1:** Colony-forming units per mL (CFU/mL) to establish the minimum bactericidal concentration (MBC) for silver sulfadiazine, aluminum chloride, copper chloride, and zinc chloride for *Escherichia coli* and *Clostridium perfringens,* and the bacteria isolated from a semen sample.

		*E. coli*	*C. perfringens*	*Biological Sample*
	*Concentration*			
AgSDZ	100 μM	-	1420 ± 1200	-
	300 μM	870 ± 1430	0 ± 0	-
	500 μM	230 ± 390	0 ± 0	>3000
	1 mM	50 ± 80	0 ± 0	420 ± 590
	10 mM	0 ± 0	0 ± 0	0 ± 0
CuCl_2_	10 mM	30 ± 30	1 ± 2	1370 ± 2280
ZnCl_2_	10 mM	1000 ± 870	3180 ± 2250	3000 ± 530

## Data Availability

The datasets used and/or analyzed during the current study are available from the corresponding author upon reasonable request.

## Supplementary Materials

The supporting information can be downloaded at: https://www.mdpi.com/article/10.3390/ijms27020773/s1.

## References

[1] Bench C., Price E., Dally M., Borgwardt R. (2001). Artificial selection of rams for sexual performance and its effect on the sexual behavior and fecundity of male and female progeny. Appl. Anim. Behav. Sci..

[2] Zak L.J., Gaustad A.H., Bolarin A., Broekhuijse M.L.W.J., Walling G.A., Knol E.F. (2017). Genetic control of complex traits, with a focus on reproduction in pigs. Mol. Reprod. Dev..

[3] Friedrichs V., Reicks D., Hasenfuß T., Gerstenkorn E., Zimmerman J.J., Nelson E.A., Carrau T., Deutschmann P., Sehl-Ewert J., Roszyk H. (2022). Artificial Insemination as an Alternative Transmission Route for African Swine Fever Virus. Pathogens.

[4] Yeste M. (2017). State-of-the-art of boar sperm preservation in liquid and frozen state. Anim. Reprod..

[5] Cheng Q., Li L., Jiang M., Liu B., Xian Y., Liu S., Liu X., Zhao W., Li F. (2022). Extend the Survival of Human Sperm In Vitro in Non-Freezing Conditions: Damage Mechanisms, Preservation Technologies, and Clinical Applications. Cells.

[6] Contreras M.J., Núñez-Montero K., Bruna P., García M., Leal K., Barrientos L., Weber H. (2022). Bacteria and Boar Semen Storage: Progress and Challenges. Antibiotics.

[7] Tvrdá E., Ďuračka M., Benko F., Lukáč N. (2022). Bacteriospermia—A formidable player in male subfertility. Open Life Sci..

[8] Althouse G.C., Lu K.G. (2005). Bacteriospermia in extended porcine semen. Theriogenology.

[9] Martín L.O., Muñoz E.C., De Cupere F., Van Driessche E., Echemendia-Blanco D., Rodríguez J.M., Beeckmans S. (2010). Bacterial contamination of boar semen affects the litter size. Anim. Reprod. Sci..

[10] Delgado-Bermúdez A., Bonet S., Yeste M., Pinart E. (2020). Long-term storage of boar seminal doses contaminated with Proteus vulgaris: A dose-dependent effect on sperm motility and sperm-bacteria interaction. Anim. Reprod. Sci..

[11] Duracka M., Lukac N., Kacaniova M., Kantor A., Hleba L., Ondruska L., Tvrda E. (2019). Antibiotics Versus Natural Biomolecules: The Case of In Vitro Induced Bacteriospermia by *Enterococcus faecalis* in Rabbit Semen. Molecules.

[12] Bussalleu E., Yeste M., Sepúlveda L., Torner E., Pinart E., Bonet S. (2011). Effects of different concentrations of enterotoxigenic and verotoxigenic *E. coli* on boar sperm quality. Anim. Reprod. Sci..

[13] Sepúlveda L., Bussalleu E., Yeste M., Bonet S. (2016). Effect of *Pseudomonas aeruginosa* on sperm capacitation and protein phosphor-ylation of boar spermatozoa. Theriogenology.

[14] Tvrdá E., Kačániová M., Baláži A., Vašíček J., Vozaf J., Jurčík R., Ďuračka M., Žiarovská J., Kováč J., Chrenek P. (2021). The Impact of Bacteriocenoses on Sperm Vitality, Immunological and Oxidative Characteristics of Ram Ejaculates: Does the Breed Play a Role?. Animals.

[15] Althouse G. (2008). Sanitary Procedures for the Production of Extended Semen. Reprod. Domest. Anim..

[16] Balouiri M., Sadiki M., Ibnsouda S.K. (2016). Methods for in vitro evaluating antimicrobial activity: A review. J. Pharm. Anal..

[17] Huan Y., Kong Q., Mou H., Yi H. (2020). Antimicrobial Peptides: Classification, Design, Application and Research Progress in Multiple Fields. Front. Microbiol..

[18] Shaoyong W., Li Q., Ren Z.-Q., Wei C.-S., Chu G.-Y., Dong W.-Z., Yang G.-S., Pang W.-J. (2019). Evaluation of ε-polylysine as antimicrobial alternative for liquid-stored boar semen. Theriogenology.

[19] Schulze M., Junkes C., Mueller P., Speck S., Ruediger K., Dathe M., Mueller K. (2014). Effects of Cationic Antimicrobial Peptides on Liquid-Preserved Boar Spermatozoa. PLoS ONE.

[20] Elmi A., Ventrella D., Barone F., Carnevali G., Filippini G., Pisi A., Benvenuti S., Scozzoli M., Bacci M.L. (2019). In Vitro Effects of Tea Tree Oil (Melaleuca Alternifolia Essential Oil) and its Principal Component Terpinen-4-ol on Swine Spermatozoa. Molecules.

[21] Ghosh P.K., Gaba A. (2013). Phyto-Extracts in Wound Healing. J. Pharm. Pharm. Sci..

[22] Vitali V., Zineddu S., Messori L. (2025). Metal compounds as antimicrobial agents: ‘smart’ approaches for discovering new effective treatments. RSC Adv..

[23] Godoy-Gallardo M., Eckhard U., Delgado L.M., de Roo Puente Y.J., Hoyos-Nogués M., Gil F.J., Perez R.A. (2021). Antibacterial approaches in tissue engineering using metal ions and nanoparticles: From mechanisms to applications. Bioact. Mater..

[24] Ning C., Wang X., Li L., Zhu Y., Li M., Yu P., Zhou L., Zhou Z., Chen J., Tan G. (2015). Concentration Ranges of Antibacterial Cations for Showing the Highest Antibacterial Efficacy but the Least Cytotoxicity against Mammalian Cells: Implications for a New Antibacterial Mechanism. Chem. Res. Toxicol..

[25] Wang L., Hu C., Shao L. (2017). The antimicrobial activity of nanoparticles: Present situation and prospects for the future. Int. J. Nanomed..

[26] Tsakmakidis I.A., Samaras T., Anastasiadou S., Basioura A., Ntemka A., Michos I., Simeonidis K., Karagiannis I., Tsousis G., Angelakeris M. (2020). Iron Oxide Nanoparticles as an Alternative to Antibiotics Additive on Extended Boar Semen. Nanomaterials.

[27] Pérez-Duran F., Acosta-Torres L.S., Serrano-Díaz P.N., Toscano-Torres I.A., Olivo-Zepeda I.B., García-Caxin E., Nuñez-Anita R.E. (2020). Toxicity and antimicrobial effect of silver nanoparticles in swine sperms. Syst. Biol. Reprod. Med..

[28] Sancho S., Briz M., Yeste M., Bonet S., Bussalleu E. (2017). Effects of the antimicrobial peptide protegrine 1 on sperm viability and bacterial load of boar seminal doses. Reprod. Domest. Anim..

[29] Waberski D., Luther A.-M. (2024). Boar semen storage at 5 °C for the reduction of antibiotic use in pig insemination: Pathways from science into practice. Anim. Reprod. Sci..

[30] Carr H.S., Wlodkowski T.J., Rosenkranz H.S. (1973). Silver Sulfadiazine: In Vitro Antibacterial Activity. Antimicrob. Agents Chemother..

[31] Haidari H., Bright R., Strudwick X.L., Garg S., Vasilev K., Cowin A.J., Kopecki Z. (2021). Multifunctional ultrasmall AgNP hydrogel accelerates healing of *S. aureus* infected wounds. Acta Biomater..

[32] Do T.B.T., Nguyen T.N.T., Ho M.H., Nguyen N.T.P., Do T.M., Vo D.T., Hua H.T.N., Phan T.B., Tran P.A., Nguyen H.T.T. (2021). The Efficacy of Silver-Based Electrospun Antimicrobial Dressing in Accelerating the Regeneration of Partial Thickness Burn Wounds Using a Porcine Model. Polymers.

[33] Ueda Y., Miyazaki M., Mashima K., Takagi S., Hara S., Kamimura H., Jimi S. (2020). The Effects of Silver Sulfadiazine on Methicillin-Resistant *Staphylococcus aureus* Bio-films. Microorganisms.

[34] Parvekar P., Palaskar J., Metgud S., Maria R., Dutta S. (2020). The minimum inhibitory concentration (MIC) and minimum bactericidal concentration (MBC) of silver nanoparticles against *Staphylococcus aureus*. Biomater. Investig. Dent..

[35] Mimura E.C.M., Favoreto J.P.M., Favero M.E., Bonifacio K.L., Peixe T.S., Morita A.A., Barbosa D.S., Yabe M.J.S., Carrilho A.J.F. (2020). Silver serum levels in burned patients treated with silver sulfadiazine and its toxicity on inflammatory cells. Burns.

[36] Olugbodi J.O., David O., Oketa E.N., Lawal B., Okoli B.J., Mtunzi F. (2020). Silver Nanoparticles Stimulates Spermatogenesis Impairments and Hematological Alterations in Testis and Epididymis of Male Rats. Molecules.

[37] Tsakmakidis I.A., Samaras T., Anastasiadou S., Basioura A., Ntemka A., Michos I., Simeonidis K., Karagiannis I., Tsousis G., Angelakeris M. (2021). Toxic and Microbiological Effects of Iron Oxide and Silver Nanoparticles as Additives on Extended Ram Semen. Animals.

[38] Cheraghi E., Golkar A., Roshanaei K., Alani B. (2017). Aluminium-Induced Oxidative Stress, Apoptosis and Alterations in Testicular Tissue and Sperm Quality in Wistar Rats: Ameliorative Effects of Curcumin. Int. J. Fertil. Steril..

[39] Elmoslemany A.M., Rehan M., Safhi F.A., Zeima N.M., El-Hassnin M.F., Elnaggar S.A., Almami I.S., Zedan A. (2024). The Antioxidant and Anti-Inflammatory Impacts of Purple and White Eggplants on Fertility and Expression of Fertility-Related Genes in Rats Treated With Aluminum Chloride. J. Toxicol..

[40] HölzleE Neubert U. (1982). Antimicrobial effects of an antiperspirant formulation containing aqueous aluminum chloride hexahy-drate. Arch. Dermatol. Res..

[41] Yaganza E.-S., Rioux D., Simard M., Arul J., Tweddell R.J. (2004). Ultrastructural Alterations of *Erwinia carotovora* subsp. *atroseptica* Caused by Treatment with Aluminum Chloride and Sodium Metabisulfite. Appl. Environ. Microbiol..

[42] Knazicka Z., Tvrda E., Bardos L., Lukac N. (2012). Dose- and time-dependent effect of copper ions on the viability of bull spermatozoa in different media. J. Environ. Sci. Health Part A.

[43] Pinto G.L., Castro J.d.S., Val A.L. (2021). Copper and cadmium impair sperm performance, fertilization and hatching of oocytes from Amazonian fish *Colossoma macropomum*. Chemosphere.

[44] Vicente-Fiel S., Palacín I., Santolaria P., Hidalgo C., Silvestre M., Arrebola F., Yániz J. (2013). A comparative study of the sperm nuclear morphometry in cattle, goat, sheep, and pigs using a new computer-assisted method (CASMA-F). Theriogenology.

[45] Jakop U., Müller K., Müller P., Neuhauser S., Rodríguez I.C., Grunewald S., Schiller J., Engel K.M. (2022). Seminal lipid profiling and antioxidant capacity: A species comparison. PLoS ONE.

[46] Ahamed M., Alhadlaq H., Khan M., Karuppiah P., Al-Dhabi N. (2014). Synthesis, Characterization, and Antimicrobial Activity of Copper Oxide Nanoparticles. J. Nanomater..

[47] Vincent M., Duval R.E., Hartemann P., Engels-Deutsch M. (2018). Contact killing and antimicrobial properties of copper. J. Appl. Microbiol..

[48] Fowler L., Engqvist H., Öhman-Mägi C. (2019). Effect of Copper Ion Concentration on Bacteria and Cells. Materials.

[49] Hu Q. (2023). Effects of zinc chloride on boar sperm quality during liquid storage at 17 °C. Veter-Med. Sci..

[50] Allouche-Fitoussi D., Breitbart H. (2020). The Role of Zinc in Male Fertility. Int. J. Mol. Sci..

[51] Björndahl L., Kvist U. (2009). Human sperm chromatin stabilization: A proposed model including zinc bridges. Mol. Hum. Reprod..

[52] Hantke K. (2005). Bacterial zinc uptake and regulators. Curr. Opin. Microbiol..

[53] Jafarirad S., Mehrabi M., Divband B., Kosari-Nasab M. (2016). Biofabrication of zinc oxide nanoparticles using fruit extract of *Rosa canina* and their toxic potential against bacteria: A mechanistic approach. Mater. Sci. Eng. C.

[54] Joe A., Park S.-H., Shim K.-D., Kim D.-J., Jhee K.-H., Lee H.-W., Heo C.-H., Kim H.-M., Jang E.-S. (2017). Antibacterial mechanism of ZnO nanoparticles under dark conditions. J. Ind. Eng. Chem..

[55] Kadiyala U., Turali-Emre E.S., Bahng J.H., Kotov N.A., VanEpps J.S. (2018). Unexpected insights into antibacterial activity of zinc oxide nanoparticles against methicillin resistant *Staphylococcus aureus* (MRSA). Nanoscale.

[56] Raghupathi K.R., Koodali R.T., Manna A.C. (2011). Size-Dependent Bacterial Growth Inhibition and Mechanism of Antibacterial Activity of Zinc Oxide Nanoparticles. Langmuir.

[57] Hernández-Sierra J.F., Ruiz F., Pena D.C.C., Martínez-Gutiérrez F., Martínez A.E., Guillén A.d.J.P., Tapia-Pérez H., Castañón G.M. (2008). The antimicrobial sensitivity of *Streptococcus mutans* to nanoparticles of silver, zinc oxide, and gold. Nanomed. Nanotechnol. Biol. Med..

[58] Maaßen I.K., Luther A.-M., Verspohl J., Waberski D. (2023). Storage of Extended Boar Semen at 5 °C Inhibits Growth of Multi-Drug Resistant *Serratia marcescens* and *Klebsiella oxytoca* while Maintaining High Sperm Quality. Antibiotics.

