# Supplementary material for: Addition of Copper Chloride and Zinc Chloride to Liquid-Stored Pig Semen Reduces Bacterial Growth Without Impairing Sperm Quality
Source: Int J Mol Sci. 2026 Jan 13;27(2):773. doi: 10.3390/ijms27020773 (PMC12840984; doi:10.3390/ijms27020773)
Supplement: Supplementary file 1 [file ijms-27-00773-s001.zip › ijms-3782739-supplementary.pdf]

Table S1. Values and standard error of the mean (SEM) for progressive and total motility in percentage for silver sulfadiazine, aluminum chloride, copper chloride, and zinc chloride at several concentrations (100 µM, 300 µM, 500 µM, 1 mM, and 10 mM) and time points (T= 0, 24, and 48 hours) after incubation at 17ºC. P value shows the statistically significant differences (P≤0.050).

		**Percentage of Progressive Motility (%)**			**Percentage of Total Motility (%)**		
		**0 hours**	**24 hours**	**48 hours**	**0 hours**	**24 hours**	**48 hours**
**AgSDZ**	**Control**	62.10± 12.49	62.00± 7.96	32.90± 32.60	87.80± 7.05	78.00± 6.72	40.20± 32.90
	**100 µM**	37.07± 29.89	0.07± 0.12*	0.56± 0.21	51.13± 37.88	3.57± 2.95*	5.53± 1.85
	**300 µM**	25.3± 27.99	0.36± 0.15*	0.20± 0.20	52.13± 38.69	4.37± 3.91*	5.13±3.00
	**500 µM**	14.70 ± 17.15	0.36± 0.47*	0.20± 0.34	23.37± 27.55	3.63±1.63*	3800± 3.40
	**1 mM**	9.50± 12.71	0.06± 0.12*	0.36± 0.47	13.53± 16.27*	2.70± 3.25*	4970± 4.64
	**10 mM**	5.73± 7.36*	0.33± 0.31*	0.40± 0.10	10.90± 10.01**	6.27±1.88**	3.93± 2.08
**AlCl3**	**Control**	62.10± 12.49	62.00± 7.96	32.90± 32.60	87.80± 7.05	78.00± 6.72	40.20± 32.90
	**100 µM**	58.40± 3.64	61.10± 10.00	45.70± 22.00	80.93± 5.12	77.10± 5.67	57.90± 22.20
	**300 µM**	68.30± 8.15	65.60± 7.30	46.80± 18.90	82.07± 3.26	73.50± 7.13	56.20± 16.70
	**500 µM**	69.20 ± 6.34	66.4± 10.50	46.4± 23.60	83.4± 6.50	74.2± 10.00	57.3± 22.90
	**1 mM**	65.63± 10.68	61.2± 6.42	49.2± 17.70	77.93± 10.85	71.5± 5.39	58.5± 16.10
	**10 mM**	48.6± 15.69	45.2± 22.40	34.7± 29.40	58.5± 11.32	54.6± 22.20	42.7± 30.80
**CuCl2**	**Control**	62.1± 12.49	62± 7.96	32.9± 32.60	87.8± 7.05	78± 6.72	40.2± 32.90
	**100 µM**	60.83± 3.44	64.7± 5.39	43.6± 35.10	79.77± 5.18	77.5± 5.67	53.3± 35.20
	**300 µM**	64.2± 15.36	67.2± 6.00	41.3± 29.80	78.83± 11.03	78.4± 5.18	51± 30.80
	**500 µM**	63.77± 5.95	61.8± 7.64	40.8± 33.00	78.83± 11.03	72± 6.06	51.2± 30.80
	**1 mM**	61.57± 8.69	60.9± 9.13	38.6± 32.30	72.97± 9.01	77.1± 4.57	49.2± 32.20
	**10 mM**	16.27± 16.74	0.26± 0.12*	0.3± 0.30	25.67± 14.98	3.77± 2.90*	7.73 ± 5.12
**ZnCl2**	**Control**	62.1± 12.49	62± 7.96	32.9± 32.60	87.80± 7.05	78.00± 6.72	40.2± 32.90
	**100 µM**	65.8± 3.83	61.7± 9.73	37± 30.20	90.43± 2.10	77.30± 9.82	46.7± 28.70
	**300 µM**	59.93± 11.70	59.2± 5.62	38.9± 33.20	86.8± 4.11	74.80 ±10.70	47.9± 29.30
	**500 µM**	67.93± 8.52	59.5± 10.80	36.5± 31.10	85.67± 12.70	73.3± 10.60	44± 28.20
	**1 mM**	61.97± 1.00	58.3± 8.65	35± 30.20	80.33± 7.55	72.6± 8.34	42.2± 29.00
	**10 mM**	37.63± 21.97	0.4± 0.35*	0.27± 0.12	49.87± 27.59	3.27± 4.82	4.93± 2.55

Table S2. Values and standard error of the mean (SEM) for Curvilinear Velocity (VCL), Straight Line Velocity (VSL), Average Path Velocity (VAP), Linearity (LIN), Straightness (STR), Wobble (WOB), Amplitude of Lateral Head Displacement (ALH), and Beat Cross Frequency (ALH) in percentage for silver sulfadiazine (AgSDZ), aluminum chloride ($AlCl_3$), copper chloride ($CuCl_2$), and zinc chloride ($ZnCl_2$) at several concentrations (100 µM, 300 µM, 500 µM, 1 mM, and 10 mM) and time points (T= 0, 24, and 48 hours) after incubation at 17ºC. P value shows the statistically significant differences ($P \leq 0.050$).

		VCL			VSL			VAP			LIN			STR			WOB			ALH			BCL		
		0 h	24 h	48 h	0 h	24 h	48 h	0 h	24 h	48 h	0 h	24 h	48 h	0 h	24 h	48 h	0 h	24 h	48 h	0 h	24 h	48 h	0 h	24 h	48 h
AgSDZ	Control	95,93 ±15,97	82,00 ±7,83	53,20 ±28,41*	95,93 ±16,53	50,10 ±7,41	35,80 ±22,60	43,53 ±6,92	67,20 ±6,27	43,40 ±23,60	65,57 ±7,46	61,80 ±12,60	62,80 ±13,10	67,83 ±18,97	75,20 ±14,30	77,20 ±14,60	68,93 ±6,71	82,00 ±1,69	81,10 ±2,15	2.96 ±0.35	2,67 ±0,30	1,63 ±1,42	9,53 ±1,70	8,40 ±0,50	5,87 ±5,08
	100 µM	55,73 ±20,44	29,70 ±4,05	26,10 ±9,76*	83,43 ±3,56	3,26 ±2,87*	6,80 ±0,53	49,23 ±9,60	15,00 ±3,31*	13,60 ±3,39	67,90 01,90±	11,70 ±11,60*	29,90 ±15,30	73,40 ±12,91	19,90 ±13,50	52,70 ±16,40	65,53 ±20,14	52,00 ±18,20	55,00 ±12,40	1,77 ±0,55	0.20 ±0,34	1,53 ±0,72	5,83 ±4,03	0,13 ±0,23**	1,03 ±1,11
	300 µM	62,60 ±5,93	29,90 ±7,42	14,40 ±1,96	93,77 ±5,94	5,40 ±2,75*	5,17 ±1,42	50,27 ±19,39	14,30 ±1,97*	9,90 ±3,74	72,30 6,03±	17,50 ±7,10*	35,80 ±6,30	77,00 ±3,48	36,60 ±16,00	53,80 ±6,29	70,86 ±12,36	48,70 ±6,21	67,50 ±17,50	2,13 ±0,35	1,30 ±0,43	0,40 ±0,36	8,00 ±0,26	0,93 ±0,32**	0,16 ±0,28
	500 µM	53,17 ±4,25	31,00 ±33,20	27,30 ±19,41*	77,07 ±28,20	4,97 ±6,62*	4,50 ±5,80	65,00 ±9,12*	12,50 ±13,70	14,00 ±9,75	68,73 ±13,20	12,60 ±5,27	12,50 ±9,00	74,06 ±4,33	31,80 ±13,11**	23,80 ±17,81	71.73 ±3,63	39,80 ±2,23	53,00 ±7,64	1,83 ±0,46	1,57 ±2,29	0,50 ±0,86	7,16 ±1,53	1,10 ±1,57*	0,16 ±0,28
	1 mM	37,30 ±23,88	12,20 ±17,60	34,20 ±27,70	87,40 ±9,42	2,57 ±2,24**	3,73 ±2,80	52,30 ±4,86	4,87 ±5,29**	15,00 ±11,11	70,50 ±7,78	32,90 ±46,40	11,10 ±9,11	61,53 ±28,50	42,00 ±43,50	24,30 ±14,00	54,43 ±18,28	43,80 ±50,40	41,40 ±12,50	1,80 ±1,05	0,20 ±0,34	0,66 ±0,61	5,63 ±4,53	0,36 ±0,66**	±0,43 ±0,40
	10 mM	45,63 ±20,44*	21,10 ±23,00	40,20 ±6,16	58,53 ±7,58	4,57 ±5,59	6,80 ±0,61	44,23 ± 7,19	9,20 ±10,11*	17,40 ±2,32	48,63 ±7,63	13,41 ±12,10	17,10 ±1,80	57,93 ±4,45	30,80 ±27,70	39,20 ±2,41	55,23 ±11,08	29,00 ±25,10	43,50 ±2,26	2,26 ±0,92	0,46 ±0,45	1,67 ±0,55	3,33 ±4,09	0,43 ±0,15*	0,66 ±0,37
$AlCl_3$	Control	95,93 ±16,53	82,00 ±7,83	53,20 ±28,40	43,56 ±6,92	50,10 ±7,41	35,80 ±22,60	65,57 ±7,46	67,20 ±6,27	43,40 ±23,60	47,16 ±16,32	61,80 ±12,60	62,80 ±13,10	67,83 ±18,97	75,20 ±14,30	77,20 ±14,60	68,93 ±6,71	82,00 ±1,69	81,10 ±2,15	2.96 ±0.35	2,67 ±0,30	1,63 ±1,42	9,53 ±1,70	8,40 ±0,50	5,87 ±5,08
	100 µM	55,73 ±19,91	70,90 ±6,09	67,20 ±17,00	29,40 ±17,49	44,80 ±6,52	42,60 ±7,33	39,20 ±22,13	61,00 ±3,32	53,20 ±14,70	54,13 ±16,11*	60,20 ±13,00	64,30 ±7,22	68,90 ±15,44	73,90 ±14,10	81,80 ±10,80	77,76 ±5,87	81,20 ±3,35	78,80 ±2,11	2,50 ±0,20	2,43 ±0,15	2,37 ±0,45	9,33 ±0,61	8,50 ±0,26	8,97 ±0,87
	300 µM	62,60 ±5,86	68,10 ±8,35	65,90 ±9,14	34,63 ±10,33	48,10 ±5,23	42,70 ±8,84	47,67 ±7,66	57,60 ±6,07	51,90 ±10,50	60,86 ±13,50	70,80 ±5,24	64,30 ±5,32	76,26 ±11,98	83,70 ±5,50	82,20 ±4,13	79,23 ±5,54*	84,60 ±1,62	78,30 ±5,39	2,5 ±0,26	2,17 ±0,321	2,33 ±0,11	9,5 ±0,60	8,83 ±0,45	8,63 0,21±
	500 µM	56,17 ±4,27	66,4 ±2,95	63,30 ±13,10	28,47 ±4,85	45,80 ±5,01	37,90 ±8,01	35,27 ±1,70	55,40 ±3,03	47,70 ±11,80	61,33 ±11,66	68,90 ±4,55	60,00 ±5,64	75,96 ±8,43	82,70 ±5,12	80,20 ±4,13	80,30 ±6,15	83,30 ±1,15	74,80 ±4,33	2,46 ±0,11	2,20 ±0,10	2,40 ±0,30	9,16 ±0,41	8,60 ±0,43	8,70 ±0,10

	**1 mM**	36,97 ±24,10	66,40 ±4,50	61,30 ±10,00	17,27 ±13,95	43,30 ±4,68	38,20 ±5.31	22,73 ±17,20	53,70 ±4,18	46,40 ±7,22	63,63 ±9,09	65,00 ±3,10	62,60 ±5,69	79,46 ±8,65	80,50 ±4,13	82,60 ±4,66	79,86 ±2,65	80,80 ±1,25	75,70 ±2,91	2,43 ±0,23	2,26 ±0,15	2,33 ±0,31	9,43 ±0,15	8,53 ±0,20	8,97 ±0,30
	**10 mM**	45,63 ±20,35	74,30 ±14,90	69,10 ±19,20	14,80 ±8,71	46,90 ±9,39	32,40 ±16,70	25,03 ±12,93	58,30 ±13,40	44,30 ±18,00	7203 ±2,15	63,10 ±0,71	44,80 ±16,50	87,73 ±1,02	80,80 ±4,03	69,90 ±11,80	82,13 ±2,43	78,20 ±3,26	62,80 ±14,70	2,40 ±0,20	2,77 ±0,32	2,90 ±0,53	8,73 ±0,05	8,03 ±0,61	8,57 ±1,34**
**$CuCl_2$**	**Control**	95,93 ±16,53	82,00 ±7,83	53,20 ±28,40	43,53 ±6,92	50,10 ±7,41	35,80 ±22,60	65,57 ±7,46	67,20 ±6,27	43,40 ±23,60	47.16 ±16,32	61,80 ±12,60	62,80 ±13,10	67,83 ±18,97	75,20 ±14,30	77,20 ±14,60	68,93 ±6,71	82,00 ±1,69	81,10 ±2,15	2.96 ±0.35	2,67 ±0,30	1,63 ±1,42	9,53 ±1,70	8,40 ±0,50	5,87 ±5,08
	**100 µM**	83,22 ±3,00	77,50 ±2,60	67,80 ±14,10	44,77 ±11,91	50,60 ±7,16	42,40 ±12,60	64,63 ±2,56	65,00 ±1,85	52,50 ±14,20	57,66 ±14,00	65,30 ±8,36	61,90 ±8,29	73,46 ±12,20	75,20 ±14,30	80.40 ±4,46	82,16 ±3,44	83,90 ±2,78	76,80 ±6,51	2,26 ±0,15	2,43 ±0,20	2,53 ±0,23	8,70 ±0,17	8,33 ±0,65	8,63 ±0,60
	**300 µM**	80,10 ±9,40	75,10 ±4,88	67,10 ±9,76	48,23 ±9,42	48,70 ±8,05	41,70 ±8,26	63,27 ±5,94	31,90 ±4,22	52,50 ±9,89	61,50 ±13,17	64,70 ±8,94	61,90 ±7,73	75,53 ±10,69	77,70 ±8,82	79,30 ±5,86	80,8 ±6,67**	82,50 ±3,10	78,00 ±5,04	2,3 ±0,17	2,43 ±0,20	2,37 ±0,31	9,10 ±0,75	8,37 ±0,35	8,53 ±0,41
	**500 µM**	80,13 ±5,45	70,50 ±0,38	60,80 ±16,30	49,53 13,01±	46,80 ±5,08	37,10 ±17,20	64,53 ±9,52	58,30 ±2,15	47,40 ±16,70	62,36 ±13,21*	66,30 ±6,82	58,30 ±15,30	77,03 ±10,10	78,30 ±8,43	75,40 ±12,40	80,43 ±6,14	82,70 ±2,74	76,50 ±8,47	2,16 ±0,28*	2,33 ±0,11	2,33 ±0,11	9,13 ±0,46	8,70 ±0,26	8,57 ±0,49
	**1 mM**	77,93 ±6,51	75,60 ±7,85	63,70 ±27,20	49,33 ±5,13	45,40 ±5,33	37,20 ±20,20	62,17 ±3,79	61,10 ±5,29	49,10 ±24,60	65,86 ±9,97	60,90 ±12,50	55,60 ±14,30	80,70 ±7,47	80,10 ±7,03	73,80 ±9,66	81,33 ±4,84	80,80 ±1,46	74,60 ±10,30	2,20 ±0,17	2,43 ±0,32	2,27 ±0,65	9,00 ±0,26	8,60 ±0,36	7,10 ±2,46
	**10 mM**	69,87 ±9,50	14,50 ±2,71*	20,80 ±13,70	50,43 ±8,23	4,43 ±1,03	8,07 ±5,54	57,50 ±9,00*	6,17 ±1,15	12,90 ±9,71	57,80 ±15,75	32,20 ±13,40	37,10 ±13,20	77,40 ±14,50	75,20 ±14,50	62,70 ±13,80	73,76 ±7,28	44,70 ±18,00	58,20 ±11,00	2,06 ±0,28	0,83 ±0,37*	0,97 ±0,87	7,46 ±0,92	0,20 ±0,34**	0,46 ±0,41
**$ZnCl_2$**	**Control**	95,93 ±16,53	82,00 ±7,83	53,20 ±28,40	43,53 ±6,92	50,10 ±7,41*	35,80 ±22,60	65,57 ±7,46	67,20 ±6,27*	43,40 ±23,61**	47,16 ±16,32	61,80 ±12,60	62,80 ±13,10	67,83 ±18,97	75,20 ±14,30	77,20 ±14,60	68,93 ±6,71	82,00 ±1,69	81,10 ±2,15	2.96 ±0.35	2,67 ±0,30	1,63 ±1,42	9,53 ±1,70	8,40 ±0,50	5,87 ±5,08
	**100 µM**	76,90 ±9,20	82,50 ±4,15	61,70 ±16,00	46,27 ±8,83	51,20 ±10,90	36.90 ±18.80	63,10 ±6,66	68,40 ±3,68	46,90 ±18,90	59,23 ±13,42	61,90 ±11,60	56,60 ±18,60	72,53 ±14,55	74,50 ±12,10	75,40 ±12,70	81,43 ±1,98	82,90 ±3,35	73,60 ±16,60	2,12 ±0,15	2,63 ±0,28	2,37 ±0,11	8,63 ±0,32	8,30 ±0,43	7,77 ±1,57
	**300 µM**	73,27 ±8,63	78,70 ±9,29	57,40 ±15,90	45,67 ±14,39	47,70 ±6,94*	34.20 ±24.60	59,50 ±10,63	64,40 ±6,180	42,40 ±22	5386 ±21,84	61,70 ±14,50	53,90 ±32,90	68,26 ±20,50	74,90 ±15,30	70.70 ±28,40	77,26 ±8,30	82,00 ±3,29	69,80 ±24,00	2,50 ±0,10	2,50 ±0,36	1,83 ±0,64	9,10 ±0,26	8,33 ±0,56	6,47 ±4,47
	**500 µM**	73,83 ±3,19	76,20 ±8,54	50,90 ±26,50	46,13 10,71±	48,20 ±2,47	33.70 ±23.20	59,40 ±5,79	63,80 ±7,19	40,00 ±25,70	67,23 ±9,87	64,10 ±9,90	59,10 ±23,50	82,80 ±9,52	76,50 ±12,00	79,20 ±12,70	55,10 ±46,06	83,80 ±1,55	72,50 ±19,60	4,63 ±3,78	2,37 ±0,20	1,73 ±0,70	7,46 ±2,40	8,47 ±0,41	6,80 ±6,56
	**1 mM**	69,50 ±7,44	75,50 ±9,32*	63,40 ±24,8	46,00 ±10,03	48,90 ±4,85	37,60 ±27,40	56,63 6,84±	62,70 ±8,36	46,50 ±27,80	60,73 ±12,40	65,20 ±7,48	52,60 ±27,70	75,36 ±16,06	78,50 ±8,51	72,60 ±21,90	61,87 ±37,38	82,90 ±2,01	69,30 ±21,20	2,33 ±0,25	2,47 ±0,20	2,10 ±0,88	9,66 ±0,72	8,60 ±0,17	7,23 ±3,25
	**10 mM**	43,47 ±1,05	17,60 ±15,30	37,40 ±7,49	25,13 ±6,92	10,50 ±9,74	5,53 ±1,85	32,10 ±3,19	12,30 ±11,11	14,90 ±0,86	75,43 ±3,92	39,60 ±36,10	15,60 ±7,18	90,96 ±2,85	56,50 ±49,10	37,00 ±11.10	82,86 ±2,40	46,50 ±41,40	40,80 ±8,37	2,10 ±0,20*	0,67 ±0,66	1,03 ±0,37	7,56 ±0,40	3,30 ±3,20	0,43 ±0,32

Table S3. Values and standard error of the mean (SEM) for viability in percentage for silver sulfadiazine, aluminum chloride, copper chloride, and zinc chloride at several concentrations (100 µM, 300 µM, 500 µM. 1 mM, and 10 mM) and time points (T= 0, 24, and 48 hours) after incubation at 17ºC.P value shows the statistically significant differences (P≤0.050).

		Percentage of Viable Spermatozoa (%)		
		**0 hours**	**24 hours**	**48 hours**
**AgSDZ**	**Control**	82.51± 3.39	85 ± 4.17	79.67± 6.03
	**100 µM**	66.6± 9.12	81.54± 5.07	48.49 ± 20.00
	**300 µM**	60.9± 7.15	74.35± 8.93	13.96 ± 16.70
	**500 µM**	64.33± 8.93	57.68 ± 28.32	4.98 ± 3.61**
	**1 mM**	68.57± 7.05	18.09± 25.83	2.71 ± 1.77**
	**10 mM**	2.19± 0.44**	3.31 ± 1.31**	3.4± 0.64**
**$AlCl_3$**	**Control**	82.51± 3.38	85± 4.17	79.67± 6.03
	**100 µM**	82.12± 5.46	85.83± 1.67	82.77± 2.22
	**300 µM**	82.99± 4.56	86.73± 3.09	84.01 ± 2.79
	**500 µM**	83.74± 3.66	86.24± 2.78	84.58± 3.70
	**1 mM**	82.02± 4.85	86.85± 2.50	83.58 ± 3.58
	**10 mM**	61.21± 7.98	66.44± 5.43	67.21 ± 4.15
**$CuCl_2$**	**Control**	82.51± 3.38	85± 4.17	79.67 ± 6.03
	**100 µM**	82.52± 5.79	85.19 ± 3.42	82.63± 3.55
	**300 µM**	83.03± 3.42	87.17± 3.54	82.52 ± 6.30
	**500 µM**	83.44 ± 4.75	86.78± 2.57	82.73 ± 4.96
	**1 mM**	83.28± 3.32	85.87 ± 4.38	83.59 ± 4.13
	**10 mM**	71.27± 1.91	47.84± 35.30	2.68 ± 1.60**
**$ZnCl_2$**	**Control**	82.51± 3.39	85.00 ± 4.17	79.67± 6.03
	**100 µM**	81.49± 4.13	84.76 ± 1.60	82.07± 4.12
	**300 µM**	79.44± 7.26	85.16 ± 3.61	82.09 ± 4.24
	**500 µM**	83.58± 3.12	85.77 ± 2.95	81.74± 4.17
	**1 mM**	84.95± 3.05	86.1± 2.05	77.17 ± 5.23
	**10 mM**	80.15± 3.24	79.21± 7.84	58.88 ± 14.80

Table S4. Values and standard error of the mean (SEM) for intracellular reactive oxygen species (ROS) in arbitrary units for silver sulfadiazine, aluminum chloride, copper chloride, and zinc chloride at several concentrations (100 µM, 300 µM, 500 µM. 1 mM, and 10 mM) and time points (T= 0, 24, and 48 hours) after incubation at 17ºC.P value shows the statistically significant differences(P≤0.050).

		Intracellular ROS (arbitrary units)		
		**0 hours**	**24 hours**	**48 hours**
**AgSDZ**	**Control**	167250 ± 126025	184110 ± 97921	194576 ± 1022645
	**100 µM**	387017 ± 32538	138645 ± 26237	177501 ± 63783
	**300 µM**	153104 ± 93772	151467 ± 61298	135227 ± 44553
	**500 µM**	95442 ± 42939	91284 ± 34427	105214 ± 47041
	**1 mM**	133836 ± 92082	80505 ± 23669	74824 ± 36463
	**10 mM**	143349 ± 120556	80731 ± 45666	40138 ± 20449
**$AlCl_3$**	**Control**	167250 ± 126025	184110 ± 97921	194576 ± 102645
	**100 µM**	203809 ± 89695	177607 ± 127086	187443 ± 127263
	**300 µM**	151826 ± 78235	156229 ± 87193	142848 ± 54806
	**500 µM**	149137± 64975	129286 ± 60245	162912 ± 122894
	**1 mM**	148486 ± 84657	168957 ± 97541	166167 ± 48700
	**10 mM**	114467 ± 38891	88747 ± 42549	114677 ± 38468
**$CuCl_2$**	**Control**	167250 ± 154348	184110 ± 97921	194576 ± 102645
	**100 µM**	203326 ± 107012	142557 ± 74104	171057 ± 91353
	**300 µM**	154272 ± 83756	156239 ± 88629	169958 ± 76347
	**500 µM**	167248 ± 93157	144769 ± 54459	168322 ± 120017
	**1 mM**	131535 ± 43267	122458 ± 50608	134009 ± 52801
	**10 mM**	138159 ± 34812	241373 ± 64734	202874 ± 172099
**$ZnCl_2$**	**Control**	167250 ± 154348	184110 ± 9792	194576 ± 102645
	**100 µM**	141153 ± 11875	118474 ± 42439	182230 ± 85896
	**300 µM**	126888 ± 28677	156837 ± 11009	165253 ± 76889
	**500 µM**	121087 ± 23171	113606 ± 30109	141840 ± 60879
	**1 mM**	136938 ± 46988	131135 ± 40854	159829 ± 52629
	**10 mM**	110093 ± 58626	112491 ± 92557	101973 ± 74026
